# Differentiating Biological Colours with Few and Many Sensors: Spectral Reconstruction with RGB and Hyperspectral Cameras

**DOI:** 10.1371/journal.pone.0125817

**Published:** 2015-05-12

**Authors:** Jair E. Garcia, Madeline B. Girard, Michael Kasumovic, Phred Petersen, Philip A. Wilksch, Adrian G. Dyer

**Affiliations:** 1 School of Media and Communication, RMIT University, Melbourne, Victoria, Australia; 2 Department of Environmental Science, Policy and Management, University of California, Berkeley, California, USA; 3 Ecology & Evolution Research Centre, University of New South Wales, Sydney, New South Wales, Australia; 4 School of Applied Sciences, RMIT University, Melbourne, Victoria, Australia; University of Sussex, UNITED KINGDOM

## Abstract

**Background:**

The ability to discriminate between two similar or progressively dissimilar colours is important for many animals as it allows for accurately interpreting visual signals produced by key target stimuli or distractor information. Spectrophotometry objectively measures the spectral characteristics of these signals, but is often limited to point samples that could underestimate spectral variability within a single sample. Algorithms for RGB images and digital imaging devices with many more than three channels, hyperspectral cameras, have been recently developed to produce image spectrophotometers to recover reflectance spectra at individual pixel locations. We compare a linearised RGB and a hyperspectral camera in terms of their individual capacities to discriminate between colour targets of varying perceptual similarity for a human observer.

**Main Findings:**

(1) The colour discrimination power of the RGB device is dependent on colour similarity between the samples whilst the hyperspectral device enables the reconstruction of a unique spectrum for each sampled pixel location independently from their chromatic appearance. (2) Uncertainty associated with spectral reconstruction from RGB responses results from the joint effect of metamerism and spectral variability within a single sample.

**Conclusion:**

(1) RGB devices give a valuable insight into the limitations of colour discrimination with a low number of photoreceptors, as the principles involved in the interpretation of photoreceptor signals in trichromatic animals also apply to RGB camera responses. (2) The hyperspectral camera architecture provides means to explore other important aspects of colour vision like the perception of certain types of camouflage and colour constancy where multiple, narrow-band sensors increase resolution.

## Introduction

The biological world is full of diversity, yet nothing attracts more ‘human’ attention than colour. This fascination is demonstrated by the amount of research exploring the mechanisms surrounding colour perception [1], the evolution of colour diversity [2], and what colours signal to receivers [3]. From an organismal perspective, as colour vision allows for the discrimination of stimuli based on spectral differences independent from brightness [4], this ability has important implications on survival and foraging. For example, colour discrimination allows a primate to identify ripe fruit [4, 5], a bee to distinguish between rewarding or mimic flowers of similar appearance [6–8], or a predator to break certain camouflage mechanisms relying on chromatic similarity between target and background [9, 10]. However, precisely matching a colour sample target is not a simple task [11].

One main principle in colorimetry is that there can be a number of different spectral signals that can result in identical stimulation of the respective colour photoreceptors, and thus lead to stimuli that are not perceivable as different. Whilst early colour theories suggested that colour perception might have been based on the eye possessing many colour sensitive cells, George Palmer [12] was the first to put forward the idea that colour perception is based on the maximal sensitivity of certain retinal particles. This idea was expanded on by Thomas Young [13] who proposed just three types of colour receptor could be responsible for our rich colour perception, an idea further developed by Hermann von Helmholtz [14] and James Clerk Maxwell [15] to create the Young- Helmholtz-Maxwell trichromacy theory [16]. An important point about the way in which a colour visual system thus processes information can be described by the principle of univariance where the output of a receptor depends upon its quantum catch, but not upon what quanta are caught [17]. One individual photoreceptor type thus pools all photons and only the resulting signal is processed by an opponent network in the brain, and therefore each individual receptor type cannot differentiate between a change in wavelength and a change in intensity [4]. This physiological principle allows for the economic production of colour monitors and commercial level cameras that use just three red, green and blue (RGB) colour channels to represent normal human colour perception [18]. For example, a calibrated monitor can provide a realistic visual representation of a complex natural environment like flowers in a garden by using just three broadband channels of information [19].

The Principle of Univariance also explains why certain spectral signals with varying spectral signatures may be perceived as being the same colour under particular illumination conditions, a phenomenon referred to as *metamerism* [4, 18, 20]. Indeed, metamerism prevents recovering a unique spectral signal directly from the response of either a biological, or human-made photoreceptor typically used in colour imaging [21–23].

The complexity of studying colour perception from behavioural responses has led to the study of animal colour vision through the physical properties of the visual signals and systems employing spectrophotometric techniques and instrumentation [24]. Although the use of spectrophotometers changed our understanding of how other non-human animals perceive different colour combinations [25, 26], the information that colour communicates [3, 27], and the evolution of colour expression [2], there is also the understanding that animal eyes do not function as point-source measuring devices. The understanding that reflectance of the object alone is not the only determinant of how colours are perceived [25, 28] is the impetus for using digital image approaches that incorporate the entire subject and its background. Reconstructing reflectance spectra from discrete pixel intensity values returned by digital imaging devices with three or more sensors has received great interest during the last fifteen years. This shift is mainly motivated by: (a) the low cost of digital cameras, (b) the convenience of sampling large and complex stimuli comprised by different colours and textures without resorting to complex sampling grids, and (c) the high amount of quality data which can be retrieved within a short time period [29]. However, to do this reliably it is vital to understand the dynamics of how different camera types process similar, or dissimilar colour stimuli.

The advantages of digital imaging have promoted the use of devices for quality control in food [30, 31], printing [32], analysis and preservation of paintings [33, 34] and other industries where colour description and specification is of importance [35]. Visual ecology and evolutionary biology have also turned their attention to the potential use of digital imaging as a tool for quantifying and characterising the spectral properties of complex, natural colour patterns such as those displayed by plants [19], and animals [29, 36, 37].

Compared to spectrophotometers, digital imaging devices with three colour channels cannot uniquely reconstruct a spectral signal due to metamerism [21]. Nevertheless, if there are enough narrow-band sensors reasonably uniformly distributed along the spectral interval effectively sensed by the camera, it is possible to uniquely recover the colour signal from the individual responses of the available colour channels. This is the rationale for recovering spectral signals from responses of hyperspectral cameras. For example, to uniquely recover a spectral signal within 400 to 710 nm sampled at 10 nm intervals, the hyperspectral system must have at least 32 separate bandwidth sensors [38].

In spite of the popularisation of the usage of digital imaging to study animal colouration, the development of spectral reconstruction methods, and the increased use of hyperspectral cameras as image spectrophotometers, there is a dearth of comparative studies exploring the potential applications of these two methods for discriminating between colours of varying chromatic similarity. Here we measure the colour discrimination thresholds of an RGB device and an hyperspectral imaging system using colour samples of varying degrees of perceptual similarity for a human observer. Due to the relationship between number of channels and the accuracy of the spectral reconstruction [21, 23], we predict chromatic similarity to be the main factor limiting the capacity of either imaging system to accurately discriminate between two colour samples [39]. The extent of such limitations is important for determining which device is optimal for different applications such as understanding floral colour signals [19] or camouflage [40, 41].

The manuscript develops as follows: A Background section introduces basic colorimetric principles and their relation to colour discrimination, followed by a formal description of the spectral reconstruction problem. This section sets the basis for explaining our spectral reconstruction algorithm which is detailed as a separate part under the Materials and Methods section, which also describes the different components of the experimental setup. The Materials and Methods section is then followed by the presentation of the results, discussion and some practical recommendations specific to hyperspectral imaging.

## Materials and Methods

### Background

#### Colorimetric principles

The description of the colour sensation produced by signals of varying spectral shape using a numerical system allows for the accurate reproduction of colours in different media such as monitors, coloured lights and pigments [18]. The development of such a system was the main goal of colorimetry, which through careful psychophysical experiments, led to the *CIE colorimetric system* currently in use [20].

The 1931 and subsequent CIE colour specification systems describe a given spectral stimulus by means of three *tristimulus values* denoted as *X, Y* and *Z*. The magnitude of these values depends on the spectral characteristics of the sample itself, the light source, and a set of functions describing the particular response of each one of the three cone photoreceptors: short, medium and long, present in the human visual system to signals of varying spectral shape [20]. These functions represent a linear transformation of the spectral sensitivity curves of the pigments present in the three photoreceptors as measured by psychophysics experiments and directly by spectrophotometry [42–45]. The tristimulus values corresponding to a given spectral signal are obtained by:

$$\begin{array}{ccc} X & = & \int_{\lambda }R\left(\lambda \right)\cdot \;L\left(\lambda \right)\cdot \;\bar{x}\left(\lambda \right)\;d\left(\lambda \right),\\ Y & = & \int_{\lambda }R\left(\lambda \right)\cdot \;L\left(\lambda \right)\cdot \;\bar{y}\left(\lambda \right)\;d\left(\lambda \right),\\ Z & = & \int_{\lambda }R\left(\lambda \right)\cdot \;L\left(\lambda \right)\cdot \;\bar{z}\left(\lambda \right)\;d\left(\lambda \right); \end{array}$$ (1)

where *R*(*λ*) denotes the spectral reflectance of the surface, *L*(*λ*) the spectral power distribution (SPD) of the light source and $\bar{x},\;\bar{y}$ and $\bar{z}$ represent functions of wavelength describing the spectral sensitivity of each one of the photoreceptors [20, 45]. In Eq (1), the product *R*(*λ*)⋅ *L*(*λ*) can be replaced by a single function *P*(*λ*), spectral radiance, rather than using measurements of the reflectance of the surface and the irradiance of the light source [20].

Eq 1 represents an industry accepted and useful simplification of the sensing process carried out by the different photoreceptor classes present in the human retina. The calculation of tristimulus values by means of this equation ignores various optical and physiological aspects of vision such as: viewing angle, spectral transmissive properties of the eye, veiling light effect as a consequence of transmission medium; i.e. air or water, and viewing distance, and assumes that the target behaves as a perfect (Lambertian) diffuse surface [20, 24]. Even though all these parameters may be accounted for by a more detailed description of the visual sensing process, the tristimulus values obtained from Eq 1 constitute the basis of the CIE colour specification system. This system is currently considered the standard for measuring perceptual colour differences between colour targets when considering human colour vision [20], and was the method followed for measuring colour differences between the selected samples.

An additional simplification in the calculation of the tristimulus values was done by replacing the continuous functions *R*(*λ*) and *L*(*λ*) by discrete wavelength intervals or bins of equal width (Δ*λ*) centred at wavelength *λ*; consequently, the integrals in Eq (1) are replaced by sums:

$$\begin{array}{ccc} X & = & \sum_{\lambda =\lambda_{a}}^{\lambda_{b}}P\left(\lambda \right)\cdot \;\bar{x}\;d\lambda ,\\ Y & = & \sum_{\lambda =\lambda_{a}}^{\lambda_{b}}P\left(\lambda \right)\cdot \;\bar{y}\;d\lambda ,\\ Z & = & \sum_{\lambda =\lambda_{a}}^{\lambda_{b}}P\left(\lambda \right)\cdot \;\bar{z}\;d\lambda , \end{array}$$ (2)

calculated over the wavelength interval visible to human observers, ≈ 390–710 nm. Bin size is selected based on the required precision of the calculations and the spectral characteristics of the employed light source, with 10 and 5 nm being the most common bin size choices [46].

#### Colour perception and discrimination thresholds

The definition and causes of the existence of colour discrimination thresholds in human colour vision are grounded on the basis of trichromacy derived from the Young-Helmholtz theory of colour perception [20, 47], and formalised by the Principle of Univariance [17]. Discrimination thresholds for large and small colour differences have been experimentally established for both simultaneous and successive colour matches in laboratory conditions using different methodologies such as colour matching [48–51] and visual search experiments [52].

The spectral signal producing a colour sensation in a given human or animal observer can be represented by a set of coordinates on a plane or *colour diagram*. A colour diagram can take into account the particular characteristics of the visual system of the observer, such as the CIE chromaticity diagram for human vision [20], or just represent a general model of colour vision as in the case of the Maxwell triangle [20, 47, 53, 54]. Irrespective of the selected colour diagram, it is often of interest to establish the minimum distance at which two or more points, each one representing a different colour sample, are perceived as being different by a given observer. As two colour samples can be different independently of their hue, e.g. red-green, red-blue, red-yellow are all perceived as being different, colour differences in a colour diagram are not represented by a single line but by a region around the selected reference point. This region determines the colour threshold for a particular stimulus but may change for other samples.

Ellipses, and circles under certain conditions, are commonly used for defining discrimination thresholds in colour diagrams [20, 54], although with modern computer techniques the representation of colour thresholds may not always be represented by classically assumed geometrical shapes. In the particular case of CIE chromaticity diagram, the colour discrimination threshold for any given sample is defined by fitting an ellipse centred at the coordinates corresponding to the reference sample which encloses all the points representing the different colour matches deemed as an identical match to the reference by the observer [55]. The size and orientation of the ellipses is typically not constant, varying in a non-linear manner with hue [48], for this reason it was necessary to empirically find the discrimination threshold for a set of 25 colours covering most of the CIE 1931 chromaticity diagram [20, 48].

In a colour matching experiment, a subject is asked to adjust the colour of a target sample of constant brightness until it matches a reference sample of known chromatic characteristics. A satisfactory colour match does not necessarily correspond to the exact chromatic properties of the sample as measured by spectrophotometry, but will be located around the reference sample. Colour samples whose chromaticity coordinates are located outside the ellipse are perceived as being different with colour difference increasing as we move away from the ellipse. The coefficients describing each one of the ellipses are not unique but vary with colour or, more precisely, with the position of the sample in the chromaticity diagram [55].

For most applications it is not necessary to reconstruct the ellipses in order to predict the perceivable colour difference between two targets. Instead, a set of contour plots describing the variation of the ellipses across the chromaticity diagram is used to recover the ellipse parameters. These parameters are then used to calculate a measure of *chromaticity difference* (ΔC) which predicts the extent at which the colour difference is easily, or just-noticeably perceivable [48]. In fact ΔC ≤ 1 represents chromaticities that are within the standard deviation of a colour match, whilst ΔC = 2 represents a *just-noticeable difference* or 1 JND. For ΔC values between 1 and 2 colour differences represent just a little less than the just-noticeable difference perceivable by the reference observer [55].

Whilst thresholds for large colour differences are useful in the construction of colour systems such as the Munsell Colour Sytem and the OSA Uniform Colour Scale [52, 56], thresholds for small colour differences form the basis of uniform colour spaces. Uniform colour spaces such as the various CIE LAB spaces constitute the basis for estimating colour differences for most industrial and consumer colour applications [18, 20]. In these colour spaces, the elliptical regions representing perceptual colour discrimination thresholds are converted into circles of equal radius. In this way, equal distances between colour samples approximately represent equally perceivable colour differences [57].

The colour difference measurement used in the uniform CIE LAB space is the ΔE metric. This metric measures colour difference in the context of the three main attributes of colour: brightness (luminance), saturation (chroma) and hue [18, 20]. These three properties describe different characteristics of the spectral signal: brightness represents the total intensity of the signal calculated as the area under the spectral curve; saturation is a measure of the steepness of the spectra within a wavelength interval; and hue is related to the wavelength of maximum slope [24].

In spite of the routine use of the various CIE LAB colour spaces and their associated measure of colour difference by the colour industry, e.g. [58], this metric has rarely tested the perception of colour difference in natural contexts. This limitation arises due to the complex visual environments where colour comparisons are commonly made, which differ to those specified for the CIE LAB colour space. For example, the specification of colour differences by the ΔE metric assumes that the colour comparison is made against a light grey to white background and assuming a ‘neutral’ reference point [20]. Moreover, the ΔE metric ignores other important aspects involved in colour perception such as the cognitive interpretation of physical properties of the object [18]. Therefore, we selected the ΔC metric over ΔE for measuring colour differences between our test targets, as the ΔC metric directly takes into account different perceptual properties involved in colour discrimination by human observers.

Except for some invertebrates [59–67] and a few vertebrate species including goldfish [68–70], mice [71], primates [72, 73], pigeons and chickens [74, 75], the precise mechanisms involved in colour perception and discrimination remain mostly unknown. For such cases several models of colour discrimination thresholds have been proposed based on purely anatomical and physiological data available from these species [24, 76–78]. However, behavioural data completely supporting or rejecting the use of these models are still missing. The application of cameras with different sensor numbers, as discussed in the present manuscript, promises to provide important insights into how to model animal colour vision.

#### Spectral reconstruction from camera responses

Eqs (1) and (2) can also be used to predict the response of a digital camera with three or more colour filters by replacing the cone photoreceptor functions by the spectral sensitivity of the system’s filters; provided that, there is a linear relationship between radiation input and camera response. This assumption does not necessarily hold for many consumer-level devices and has to be tested prior to reconstructing spectral signals [29, 79]

The function *L*(*λ*) in Eq (1) describes the SPD of any light source illuminating the sample. Examples of light sources include technical lamps [18], as well as for the different conditions of daylight [80] or the ambient light reaching the floor under the canopy in a forest [24]. Values for *L*(*λ*) are available for different lamps [18], terrestrial and aquatic environments at different times of day and conditions [81, 82]. On the other hand, the values for the *R*(*λ*) or *P*(*λ*) functions must be measured directly from point samples on the coloured object either by using a spectrophotometer or a spectroradiometer [24, 83].

Due to the practical difficulty of accurately measuring *R*(*λ*) or *P*(*λ*) from point samples on natural patterns presenting high spatio-chromatic variability due to texture and volume [19], it would be desirable to recover the values for these functions directly either from camera or photoreceptor responses; in other words, solving for *R*(*λ*) or *P*(*λ*) by inverting Eq (2). When camera responses are used for this end, the process is referred to as *spectral reconstruction*.

The rationale for spectral reconstruction is more easily understood by expressing Eq (2) implementing matrix notation [34, 84]:

$$\begin{array}{c} p=\left(SE\right)r+\epsilon , \end{array}$$ (3)

where **p** is a *p* × 1 vector holding the camera responses for each of the *p* sensors present in the system, **E** is a *m* × *m* square matrix holding the values of *L*(*λ*) in its diagonal, i.e. **E** = *diag*(*L*(*λ*)), **S** is a *p* × *m* matrix holding the spectral sensitivity functions for each one of the *p* sensors present in the system measured in *m* equally-spaced wavelength bands, **r** is a *m* × 1 vector holding the reflectance values for the recorded sample and ***ϵ*** is a *p* × 1 additive noise vector. For example, for an RGB camera with red, green and blue sensors and with spectral sensitivity functions measured at Δ*λ* = 10 nm intervals within a 400 to 710 nm spectral range, *p* = 3 and *m* = 32.

Even in the ideal system without noise or where it could be perfectly modelled, the dimensionality of **S** limits the accuracy of the spectral reconstruction attained by inverting Eq (3). Continuing with the example of the RGB camera, the **SE** matrix would be a 3 × 32 matrix resulting in a an equation system with 32 unknowns in 3 equations. Such a system is undetermined with infinite possible solutions [85]. There are two strategies for improving the accuracy of the spectral reconstruction of the signal’s spectrum: a) reducing the dimensions of **SE** to match those of **p**, or b) increasing the number of responses in **p** such that it matches the dimensions of **SE**.

The dimensions of the **SE** can be reduced by including *a priori* information about known properties and characteristics of reflectance spectra of natural and human-made colour surfaces [85–87]. Dimension reduction is achieved by expressing the spectral functions in Eqs (1) and (2) as linear models with a reduced number, typically 6 to 12, of basis functions [86–90]. Likewise, the **SE** matrix in Eq (3) can also be replaced by a matrix that relates the spectral sensitivity functions of the camera to the reflectance spectra of a set of calibration samples of known spectral properties or *calibration set* [86]. Alternatively, it is also possible to relate the camera responses to the reflectance spectra of the calibration set directly [88]. *A priori* information can also be used to accurately estimate the noise parameter ***ϵ***. In fact, among the most recent spectral reconstruction methods are those based on the characterisation of the noise vector either by direct or analytical methods and subsequent reconstruction of the reflectance spectrum by optimisation techniques [85, 91, 92].

When reflectance spectra are reconstructed from camera responses using linear models, the number of camera channels should match the number of basis functions. However, as just three basis functions are not enough for accurately reconstructing a spectrum, the three channels available in an RGB camera are potentially insufficient for recovering the the signal spectrum with any of these methods [90]. Nevertheless, it is possible to increase the number of camera responses from these devices by sequentially recording a set of images through different narrow-band filters of varying spectral bandwidth [38]. This effectively converts an RGB device into a *multispectral camera* [93]. For example, commercial-level RGB cameras can be fitted with three additional narrow-band filters thus increasing the size of **p** from 3 × 1 to 6 × 1 for reconstructing spectral signals from natural scenes [94].

An alternative method for reconstructing spectral signals by means of RGB devices is by recovering the *metamer set* [21, 95]. The goal of this approach is to recover not the single reflectance spectrum responsible for a given RGB response, but the set of all spectra that can produce an exact match to it [23]. Recovering the metamer set for a given RGB response can be achieved with or without knowledge of the camera’s sensor spectral sensitivity [21, 23]. In the latter case, the recovery of the set is achieved by exploiting the convex properties of both the RGB and the spectral space. Convexity permits expressing any reflectance spectrum as the linear combination of a set of calibration spectra comprised of primary colours with high brightness and saturation values. The weights that must be applied to the calibration spectra to match the sample spectrum are the same as those which relate the RGB values of the calibration spectra to those of the unknown sample [23]. Therefore the reconstruction of the metamer set is based on finding these weights from the RGB responses of the samples in the calibration set and subsequently applying these values to the reflectance spectra available in the calibration set. Mathematically this is expressed as [23]:

$$\begin{array}{ccc} p & = & P\lambda ,\;\text{subject}\;\text{to}\\ 1^{T}\lambda & = & 1,\\ I\lambda & \geq & 0,\\ I\lambda & \leq & 1; \end{array}$$ (4)

where **P** is a 3 × *n* matrix holding the RGB responses of a calibration set of *n* samples, and **λ** is a *n* × 1 vector of weights, *n* being the number of spectra in the calibration set.

Our spectral reconstruction methodology is also based on the recovery of the metamer set for a given sample. In contrast to the method proposed in [23], we find the weights vector **λ** by testing all the possible linear combinations of four RGB points available in the calibration set **P**, and subsequently identifying those combinations of RGB responses for which the sample RGB is an internal point. In other words, we search and test for all the RGB points whose convex combination leads to an RGB specification equal to that of the sample. This method contrasts with the one in [21, 23] where the weights vector is obtained by intersecting the hyperplanes representing the constraints in Eq (4). Further details of our spectral reconstruction methodology are provided in the Spectral Reconstruction subsection.

A more expensive alternative to using a filter-fitted RGB camera is using a hyperspectral camera. This device records the reflectance spectrum of a given sample at each pixel location within the recorded image directly by inverting Eq (3). This is possible as the number of available camera responses is equal or higher than the dimensions of the matrix **SE** [38]. For example, recovering the reflectance spectrum from a coloured object sampled within the 400 to 710 nm interval at Δ*λ* = 10 nm would require a hyperspectral system with a minimum of 32 sensors. Spectral reconstruction from hyperspectral images does not require any *a priori* information from the reflectance or the illumination spectra, the only information required is the spectral sensitivities of the system’s sensors, noise statistics and a linear relationship between energy input and camera response [96]. Therefore, this system is expected to produce the most accurate reconstruction of the sample’s reflectance spectra; in fact, hyperspectral cameras are also referred to as image spectrophotometers [38]. The main drawback of these devices is the long total integration time required to record the hyperspectral image cube (hypercube), resulting from the sum of the individual integration times required to record the image at each wavelength step.

### Target colour sample pairs and calibration set

**Fig 1:**
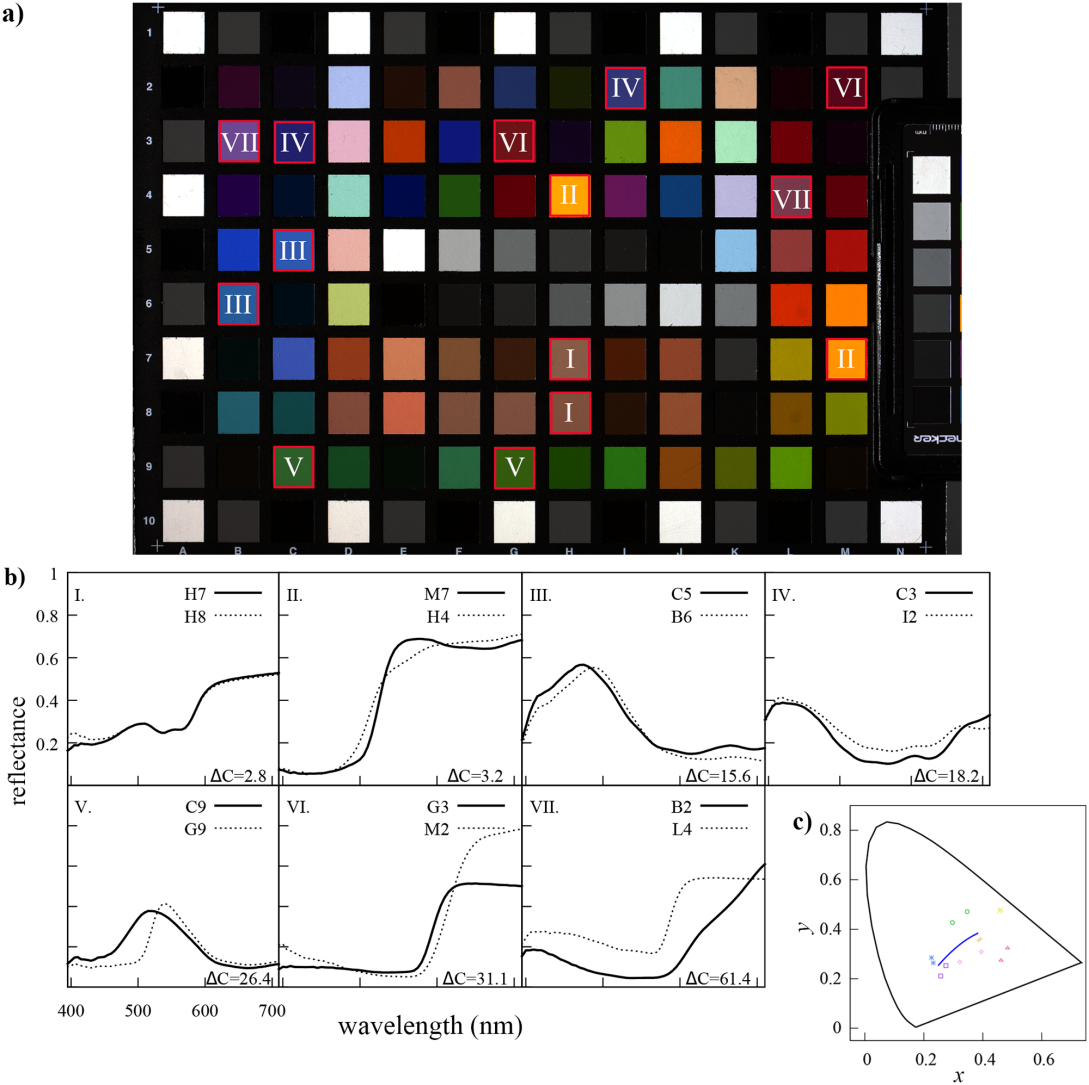
Colorimetric characterisation of the selected target colour pairs. a. Linear RGB representation of the X-rite ColorChecker SG highlighting the seven colour pairs selected for the experiment. The samples comprising each colour pair are identified by the same roman numeral. b. Reflectance spectra for the 14 individual colour samples making up each one of the colour pairs and their chromaticity difference values (ΔC). Chromaticity difference values were calculated from chromaticity coordinates (Table 2) using formulae and diagrams by MacAdam [48] and assuming a mercury discharge lamp illumination. Individual colour samples are identified by their unique coordinates in the chart (panel a). c. Colour samples in the 1931 CIE chromaticity diagram: (+) pair I, (×) pair II, (*) pair III, (◻) pair IV, (○) pair V, (△) pair VI and (⋄) pair VII. Colour key of each pair is a crude representation of the linear RGB combination for each pair in panel a.

We selected seven pairs of colour samples available in the Digital ColorChecker SG (X-rite Inc., USA) varying in chromaticity difference values (ΔC) [48] from 2.8 up to 61.4 units (Fig 1). Chromaticity difference (ΔC) values between the two colours making up each of the seven pair samples were obtained from the coefficient describing the MacAdam’s ellipses for these samples. Ellipse coefficients for each pair of samples were obtained from published graphs [48] after calculating the chromaticity coordinates corresponding to the tristimulus values for each colour sample (Eq 1) following standard colorimetric calculations [20].

Samples were selected in such a way that they: (a) represent different colour hues: orange, red, yellow, blue, green, pink and violet, (b) represent different amounts of perceptual chromatic dissimilarity in terms of just noticeable differences [20, 48], and (c) did not represent extreme points (vertices) in the RGB linear space defined by the samples available in the calibration set.

Our calibration set contained a total of 1393 colour samples corresponding to the 1301 chromatic and achromatic samples available in The Munsell Book of Color Matte Collection (X-rite Inc., USA), plus the 94 colour samples available in the Digital ColorChecker SG after removing the samples corresponding to each target sample pair. These carefully specified samples are based on pigments and have been used in previous colour experiments [97, 98], representing a broad range of hues. Whilst the stimuli lack of UV components that are important for some animal vision and cannot therefore represent spectral characteristics of all natural spectra, the stimuli broadly have spectral characteristics consistent with the shape and gradients of some natural stimuli like flowers [99] when considering wavelength greater than 400 nm (see on-line data files). The 44 achromatic samples bordering the Digital ColorChecker SG (Fig 1, panel a) were not included as part of the calibration set. Details on the composition of the calibration set and each one of the test targets are available in Table 1.

**Table 1:** Details of the composition, source and number of colour samples included as part of the calibration set and target colour sample pairs.

	Calibration set	Target colour sample pair
Source	Munsell Book of Colour & X-rite	X-rite
Number of samples	1301 (Munsell) + 92 (X-rite)	14 colour samples in 7 pairs
Sample details	Red: 139 samples in 4 hues	Pair I: H7 & H8
	Yellow-Red: 123 samples in 4 hues	Pair II: M7 & H4
	Yellow: 143 samples in 4 hues	Pair III: C5 & B6
	Green-Yellow: 127 samples in 4 hues	Pair IV: C3 & I2
	Green: 115 samples in 4 hues	Pair V: C9 & G9
	Blue-Green: 106 samples in 4 hues	Pair VI: G3 & M2
	Blue: 112 samples in 4 hues	Pair VII: B3 & L4
	Purple-Blue: 137 samples in 4 hues	
	Purple:131 samples in 4 hues	
	Red-Purple: 137 samples in 4 hues	
	Neutrals: 31 samples in 8 Values	
	Xrite: 92 samples^†^	

^†^Excludes the two samples used as target colour pair for each test.

Reflectance spectra from the target colour pairs and the calibration set were measured within a 390 to 710 nm interval with an USB 4000 spectrophotometer (Ocean Optics, USA) connected to an ISP-50-8-R-GT integrating sphere (Ocean Optics, USA) by means of a 200 *μ*m optical fibre (Ocean Optics, USA). The halogen lamp of a DH-2000 light source (Ocean Optics, USA) was used as illumination source, and connected to the integrating sphere by means of a 600 *μ*m optical fibre arranged in a 0/*d* illuminating and viewing condition [20]. Reflectance values were measured relative to a certified Spectralon reflectance standard (Labsphere, USA).

**Fig 2:**
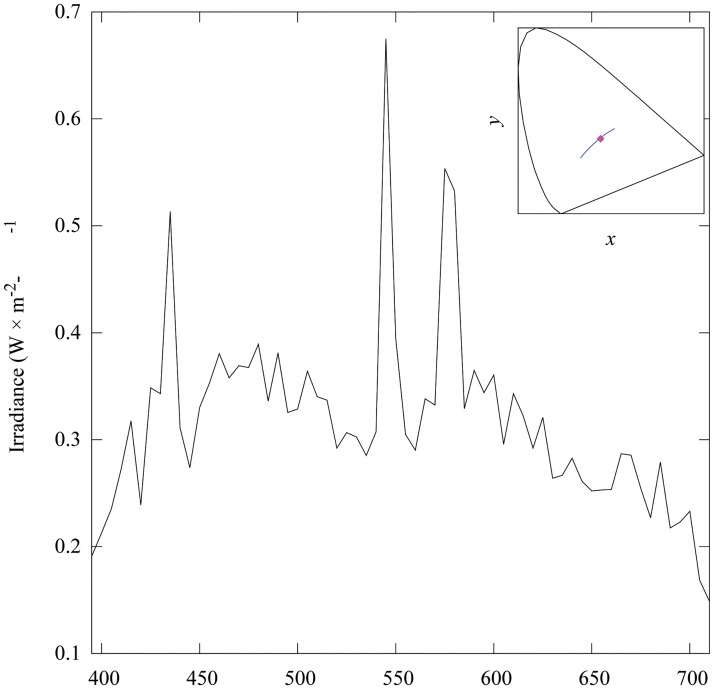
Spectral power distribution (irradiance) and chromaticity of the mercury discharge lamp used as light source for the experiment. Chromaticity coordinates corresponding to the light emitted by lamp (insert) were calculated from tristimulus values obtained after solving Eq (2) using the CIE 1931 colour matching functions [20]. Spectral irradiance from the light source was measured with an ILT 900 spectroradiometer (International Light Technologies, USA), calibrated for irradiance measurements. Blue solid line in the insert represents the CIE daylight locus for correlated colour temperatures between 4000 to 25000 K [80].

Chromaticity values corresponding to each of the sample pairs (Fig 1, panel c) were calculated from their tristimulus values obtained after solving Eq (2). Calculations were made considering a 390 to 710 nm spectral range sampled at Δ*λ* = 5 nm bins. The $\bar{x},\;\bar{y}$ and $\bar{z}$ functions in Eq (2) correspond to those of the 1931 CIE colour observer [20]. The spectral power distribution of the light source in Eq (2) (Fig 2) was taken to be the irradiance emitted by a Broncolor HMI 400.575.800 mercury discharge lamp (Bron Elektronik AG, Switzerland) used to illuminate the Digital ColorChecker SG when recording images with the two camera systems.

The HMI discharge lamp was connected to an electronic ballast providing a stable current supply at 400 Hz thus minimising potential flickering effects. The mercury lamp emitted a radiation with similar chromatic characteristics to those of daylight (Fig 2, insert) with a high output of short (390–450 nm) wavelength radiation when compared to a halogen tungsten lamp. The availability of short wavelength radiation was of particular advantage for our purposes as it helped to diminish the high noise levels expected from the low sensitivity of both camera systems to this type of radiation.

### Camera system and image recording

Reflectance spectra from the colour samples comprising each one of the target pairs were recovered from digital responses, *i.e.* pixel counts, recorded by two camera systems: (a) a calibrated Canon EOS 40D RGB digital camera (Canon Inc., Japan) with 3 channels and (b) a SOC710 hyperspectral imaging system (Surface Optics Co., USA) with 128 channels (bands). The RGB camera was equipped with an 100 mm, electro-focus (EF) macro lens (Canon Inc., Japan) with a maximum *f*-stop of 5.6. The RGB camera system has been previously characterised for: (a) recovering of linear camera responses by analytical means and look-up tables, (b) quantification of the uncertainty associated with the linearisation process for camera responses of varying intensity [79], and (c) to quantify total radiation reflected by various flower species [19].

The SOC710 imaging system was equipped with a 70 mm Schneider Xenoplan lens (Schneider Optics, USA) with a maximum aperture of *f*/8. The hyperspectral camera utilises a 696 × 520 pixel CCD sensor sensitive to radiation between 400 to 1000 nm. Individual images are produced for each one of the 128 different bands covering the spectrum, with a dynamic 12-bit range [100]. The camera was connected to a PC laptop and operated by means of *Lumenera* drivers v.6.3.0 provided by the manufacturer. The 40D Canon camera has a 3888 × 2592 pixel CMOS sensor, with a measured spectral sensitivity limited to the visible region of the electromagnetic spectrum (about 390 to 710 nm) [19]. The camera records images in a 14-bit colour depth when shooting in RAW mode.

The Digital ColorChecker SG chart was aligned at 45° with respect to the sensor plane of each camera preventing any specular reflection produced by the semi-glossy surface of the chart from reaching the sensor in both cameras, and the light source was oriented at 45° with respect to the ColorChecker. Two Munsell grey N5/stripes 8 mm width were attached to the lateral borders of the ColorChecker SG chart as an extra achromatic reference.

The colour chart was centered on the viewfinder of the RGB camera, and on the focusing window available as part of the hyperspectral camera control software. The target was recorded with one camera at a time to prevent potential differences in illumination produced by changes in the lighting geometry.

The hyperspectral camera total integration time was determined at 10.00 ms by the camera’s onboard light metering system. The integration time selected by the hyperspectral camera summed up the exposure time required by all the channels as our camera model and operating software did not allow for setting up the integration time individually for each channel. The exposure for the RGB camera was set at 1/640 s, *f*-stop 8, ISO 200 after performing several exposure tests.

Variability in the DSLR camera responses, which could be potentially introduced by unstable light source output, or the mechanical components of the DSLR camera such as shutter curtain and lens aperture blades, was measured in a separate control experiment. For this experiment we initially recorded, under nominally identical conditions, a series of 30 images of a set of achromatic targets of varying brightness. Then we selected a 25 × 25 pixel area corresponding to a mid grey and calculated the mean pixel intensity response for the sample area on each frame. Mean intensity response for the same target on each image was then plotted against image number to measure any potential variability in camera responses across the 30 shots. Variability was measured as the absolute difference between the mean minimum and maximum mean intensity value across all the recorded frames. We obtained a mean intensity of 162 intensity levels across the 30 images and minimum and maximum values of 159 and 164 intensity levels respectively. This result shows that the variability in camera response introduced by the system is around ±2 intensity levels, which represents a variation of about 1% in an 8-bit scale.

### Image Processing

#### RGB imaging system

RGB images from the DigitalChecker SG colour chart were recorded in the native RAW format of the Canon camera using the 5100 K white balance colour setting. To ensure a correct exposure and white balance setting in the linearised images, several versions of the original RAW image were produced varying the exposure, colour temperature and tint values of the original, unprocessed RAW image. This was an iterative process where several uncompressed TIFF file versions of the original RAW file were produced after encoding each one of the RAW processed image versions into the Adobe 1998 RGB 8-bit colour space [101]. Exposure, white balance calibration and image conversion was done using the Camera Raw v.7.3 plug-in for PhotoShop CS6 (Adobe Inc., USA).

We used the RGB responses corresponding to brightest achromatic patch of an X-rite Colorchecker Passport (X-rite Inc., USA) included on each frame (Fig 1) as an exposure calibration reference. An image was deemed as properly calibrated when the mean camera responses (*ρ*) for the achromatic calibration patch were equal to 244 pixel intensity levels across the three camera colour channels or ${\bar{\rho }}_{R}={\bar{\rho }}_{G}={\bar{\rho }}_{B}=244$. A pixel response of 244 intensity levels in our camera system corresponds to the measured 94.8% reflectance value of the achromatic calibration sample after linearising the image [19, 102].

Each one of the images containing the RGB responses of the calibration set for the spectral reconstruction process were processed following the protocol just described. A total of 43 images containing the different pages of the Munsell Book of Color matte edition were recorded under the same light source used to record the ColorChecker SG colour chart (target image). To ensure that both the calibration and target images shared the same exposure and colour balance properties, a robust within-subject ANOVA was performed on the RGB responses corresponding to the brightest achromatic target of the X-Rite Colour Checker Passport. Mean camera responses for the red (${\bar{\rho }}_{R}=244\pm 0.125$), green (${\bar{\rho }}_{G}=244\pm 0.121$) and blue channels (${\bar{\rho }}_{B}=244\pm 0.054$) were obtained for the achromatic calibration target across the 44 images after exposure and white balance processing. These values were not statistically different from each other (*P* = 0.795). The final exposure and white balance parameters for the target image were set as: colour temperature 5480 K, tint -6, exposure as shot.

Colour-corrected images were subsequently linearised using look-up-tables (LUT) derived for the employed camera using custom code written for Matlab release 2012b (The MathWorks, USA). Details of the linearisation methodology and a description of the entire camera characterisation process are available elsewhere [79]. The 1395 linear RGB values used for the spectral reconstruction exercise were obtained after bootstrapping the mean linear camera responses of a 2500 × 2500 pixel sample area located at the centre of each colour sample.

All statistical analysis including: bootstrapping, ANOVA tests, linear and non-linear regressions were done using routines available in the R Statistical Language v.3.1.0 [103].

#### Hyper spectral imaging system

The hyperspectral image cube (hypercube) containing camera responses for each of the 128 channels recorded by the imaging system was initially calibrated to express camera responses in radiance units (mW ⋅ cm^−2^ ⋅ nm^−1^ ⋅ sr^−1^). The image cube calibration was carried out using a dark image reference file recorded immediately after photographing the target, the calibration file provided by the manufacturer and the integration time required to record the ColorChecker SG. The recorded radiance-calibrated image hypercube consisted in a 520 × 696 × 128 stacked matrix, where each one of the 128 dimensions corresponds to a 520 × 696 monochrome image resulting from recording the scene through the different bands available on the system. A reduced hypercube containing images from the spectral bands 390.2 to 721.1 nm at Δ*λ* = 4.9 nm intervals was exported into Matlab release 2012b (The MathWorks Inc., USA) for further processing.

Ten pixel locations within the image area corresponding to each colour sample were pseudo-randomly selected for analysis. Each pixel sample was represented as a 66 × 1 vector whose entries correspond to the radiance recorded at each one of the wavelengths sensed by the different channels of the hyperspectral system. Radiance values within the 395 to 710 nm spectral interval at Δ*λ* = 5 nm were recovered by linear interpolation. This step was necessary in order to match the sampling interval of the $\bar{x},\;\bar{y}$ and $\bar{z}$ colour matching functions, and the spectral irradiance measured for the mercury discharge lamp used as light source (Fig 2). This was the same spectral irradiance used for the calculation of the chromaticity values from the measured reflectance spectra. Tristimulus values and their corresponding chromaticity coordinates were calculated for each interpolated radiance spectrum following the same protocol as for the measured reflectance spectra.

A second hyperspectral image cube expressing camera responses in terms of absolute reflectance at each pixel location was also reconstructed from the raw hyperspectral data. The calibration process for obtaining reflectance data was the same as that described above for reconstructing the radiance spectra, but included an additional light reference calibration step. The light reference consisted of a pixel sample area corresponding to a spectrally flat (achromatic) sample area within the recorded scene. We selected an image area corresponding to the ‘white’ patch of the ColourChecker SG (sample E5 Fig 1, panel a.). Absolute reflectance data for each sampled pixel were then recovered by dividing each radiance spectra by the light reference spectrum. This calculation was done automatically by the hyperspectral camera analysis software. The remainder of the sampling methodology was identical to the one implemented for the reconstructed radiance spectra.

### Spectral reconstruction from RGB camera responses

Our spectral reconstruction system is based on the convex properties of both RGB and spectral spaces [23]. The reconstruction algorithm begins by enumerating the (nk) combinations of *k* = 4 points that can be obtained from each one of the *n* RGB coordinates corresponding to the samples available in the calibration set. The **λ** weights vector for each four point combination is then obtained from Eq (4) by left division with the additional constraint that the sum of the *λ*_*i*_ weights must equal one. If all coefficients are non-negative, the weights are included in the output vector, otherwise that weight vector is discarded and the algorithm proceeds with the next RGB combination.

**Fig 3:**
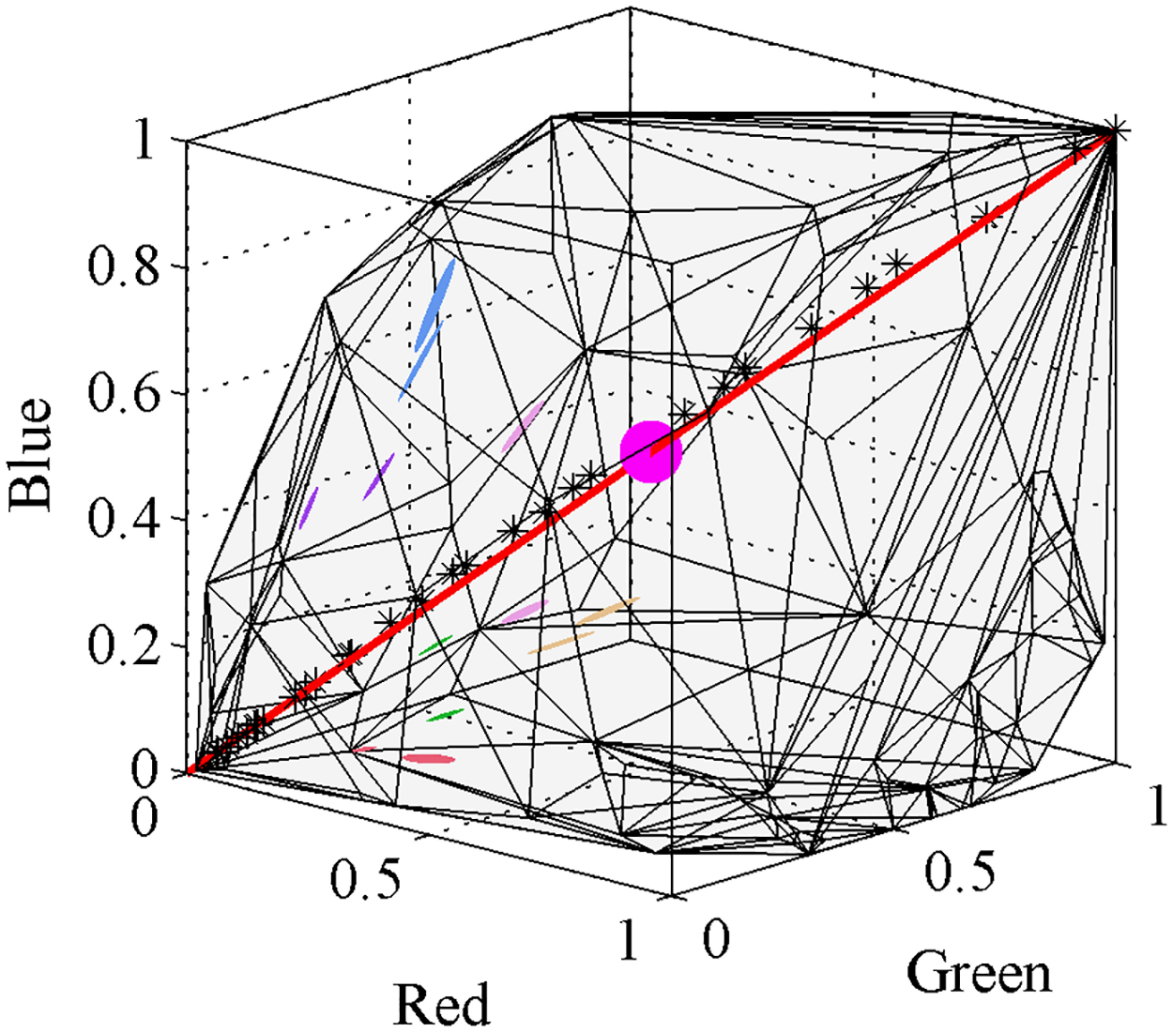
Convex hull in linear RGB space corresponding to the 1395 samples available in our calibration set, and 95% quartile ellipsoids from the 10 pixels sampled from each colour sample. Colour samples constituting a pair are coded using the same colour and code as in Fig 1: pair I pale orange, pair III blue, pair IV purple, pair V green, pair VI red and pair VII pink. Pair II are not ellipsoids but lines as these samples only present variation along the green axis. The red line represents the theoretical RGB responses for a range of spectrally uniform, achromatic samples (*ρ*_*R*_ = *ρ*_*G*_ = *ρ*_*B*_) varying in brightness, and the asterisk markers represent measured linear camera responses (*ρ*) for the 31 achromatic samples available in the Munsell Book of Colour. The magenta sphere indicates the center of the hull corresponding to a theoretical, spectrally flat, achromatic sample reflecting 50% of incident radiation.

A valid weights vector **λ** is only obtained when the RGB response of the sample can be expressed as a positive combination of RGB responses from the samples in the calibration set. Geometrically this means that the sample is located within the closed tri-dimensional space formed by the linear RGB values of the calibration set, or more precisely, when the sample is within the convex hull (ch) defined by the set *S* of linear RGB values corresponding to the calibration samples. If a sample is not within the convex hull, it constitutes a vertex of the hull; no solution can be obtained for vertices with our algorithm. Fig 3 shows the convex hull corresponding to the 1395 samples available in the calibration set and the 95% quartile ellipsoids corresponding to the 10 pixels sampled from each colour target.

Attempting spectral reconstruction with our algorithm using all the samples available in the calibration set is unfeasible in most standard computers due the large number (1.57 × 10^11^) of possible combinations. Therefore, we selected a subset of 100 calibration points from our entire set for reconstructing the spectra of each target. A calibration set of about 100 samples has been previously proposed as the minimum size at which accuracy of the reconstruction becomes independent from sample size in multispectral systems [35], and provides a number of combinations (≈ 3.92 × 10^6^) that is manageable by most standard computers.

We selected each particular subset of calibration samples based on their relative distance to the sample in the linear RGB space. For each sample, we calculated its Chebychev distance to each of the other samples in the calibration set, and selected the 100 closest points to the sample as the reference set. The Chebychev distance examines the distance between the target and the calibration targets across the three RGB channels but only uses the maximum distance [104]. This process thus ensures creating subsets of similar colour. Spectral reconstruction from reference samples of a colour similar to that of the target has been shown to improve the accuracy of the reconstruction when using methods based on basis functions [105, 106]. We also included the white and black samples in each calibration subset to ensure that it was properly ‘closed’ at its extreme points (Fig 3).

### Evaluation of spectral reconstruction accuracy and chromatic discrimination power

Accuracy of the spectral reconstruction from camera responses was measured in terms of perceptual colour differences between the chromaticity values calculated from reflectance spectra measured with a spectrophotometer and those reconstructed from the hyperspectral and RGB camera responses.

Chromaticity values corresponding to the measured and reconstructed reflectance spectra were calculated from tristimulus values (Eqs 1–2) using standard colorimetric formulae [20]. Due to the large number of reflectance spectra reconstructed from a single RGB camera response, and the non-normal distribution of their corresponding chromaticity coordinates in the 1931 CIE chromaticity space (see Results section), we expressed our results as the area delimited by the convex hull enclosing all the chromaticity values rather than by ellipses such as those produced from applying (parametric) multivariate measures of location and spread. The area in the 1931 CIE chromaticity diagram enclosed by the convex hull of a the chromatic coordinates corresponding to the reconstructed metamers of a colour sample are denoted as *chromaticity areas* (CAs).

In the case of the metamer sets reconstructed from the RGB camera, chromaticity coordinates within any given CA represent colour signals indistinguishable from one another; therefore they are partially equivalent to the discrimination ellipses previously used by MacAdam and others for measuring chromatic discriminability in simultaneous colour-matching experiments [48–51]. However CAs do not represent a region containing a specific percentage of accurate discrimination events, but the absolute colour discrimination threshold of the RGB camera given the available calibration set. The vertices making up the CAs were calculated using the *convexHull* algorithm available in Matlab release 2012b (The MathWorks, USA). CAs were also calculated from the chromaticity values obtained from the radiance and smoothed reflectance spectra reconstructed from the hyperspectral image cube for comparison.

The final experiment compared the two imaging systems in terms of their capability to discriminate two colour samples of varying colour similarity. The power of each imaging system to accurately discriminate two colour samples was measured in terms of the amount of overlap between the CAs corresponding to colour samples making up each one of the selected sample pairs (Fig 1). The intersect region between two CAs is here referred to as the *area of confusion region* (ACR). We calculated ACRs with the algorithm for polygon operations *polybool* available in the mapping toolbox v.3.6. for Matlab release 2012b (The Mathworks, USA).

We tested for a possible significant effect of colour similarity on chromatic discrimination power by fitting a logistic regression model. The model was fit to a plot of percentage of ACR for a given CA as the dependent variable and colour similarity measured in terms of chromatic difference (ΔC) as the independent variable. For this model, we used the generalised linear modelling algorithm (GLM) available for the R Statistical Language v.3.1.0 [103].

## Results

### Spectral reconstruction with RGB and hyperspectral imaging systems

**Fig 4:**
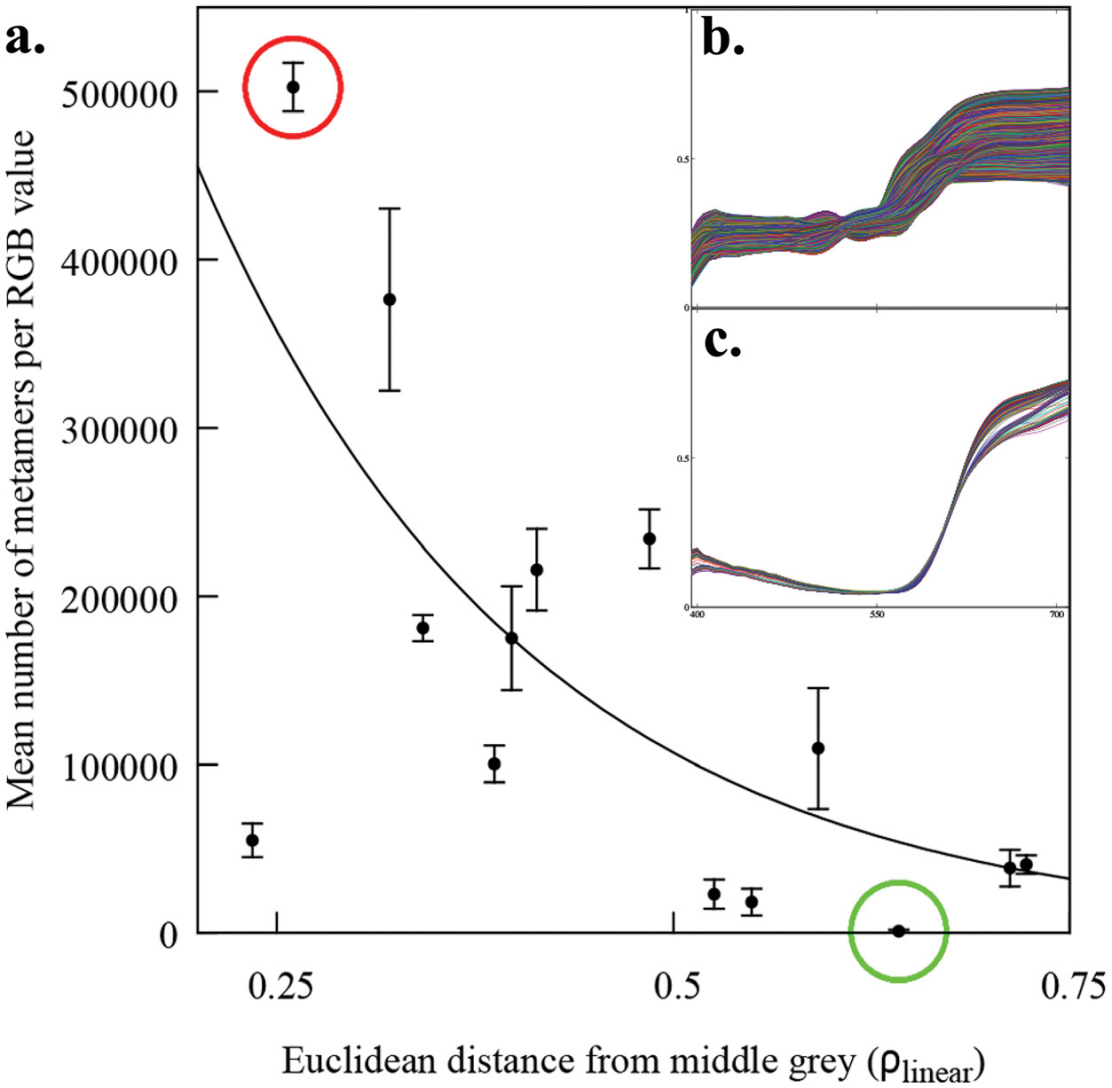
Mean number of metamers recovered from a given *ρ*_*R*_, *ρ*_*G*_, *ρ*_*B*_ triplet camera response as a function of distance from the mid point of the RGB linear space. In panel a. each point corresponds to the mean number of metamers recovered for each *ρ* triplet and the error bars represent standard deviation from the mean. Solid line represents the best fit of an exponential model of the form *y* = *y*_0_ exp−(*bx*) (Eq 5) fitted by means of a least absolute deviation (LAD) regression [107]. b. Metamer set (477,341 metamers) for *ρ*_*R*_ = 0.508, *ρ*_*G*_ = 0.327, *ρ*_*B*_ = 0.246 located at 0.307 linear RGB units from the center (red circle in a.) corresponding to a pixel sample from colour target H8 in Fig 1. c. Metamer set (483 metamers) for *ρ*_*R*_ = 0.337,*ρ*_*G*_ = 0.025,*ρ*_*B*_ = 0.097 located at 0.644 linear RGB units from the center (green circle in a.) corresponding to a pixel sample from colour target M2 in Fig 1.

We reconstructed the reflectance spectra from ten pixel sample points for each one of the 14 colour targets making up seven selected pair samples varying in colour difference (Fig 1) using an RGB and a hyperspectral camera system. Whilst the hyperspectral camera system always produced one reflectance spectrum per sampled pixel, a set of various reflectance spectra (the metamer set), was always obtained for any RGB camera response triplet (***ρ***). The total number of reflectance spectra making up a given metamer set was not constant but was found to be significantly correlated with the sample’s position within the convex hull produced by the calibration samples (Eq 5). As the Euclidean distance between a sample’s ***ρ*** and the the mid-grey central point (*ρ*_*R*_ = *ρ*_*G*_ = *ρ*_*B*_ = 0.5) increases (Fig 3), the number of metamers (N_metamers_) decreases in an exponential fashion (Fig 4):

$$\begin{array}{c} {\text{N}}_{\text{metamers}}=\exp\left[-4.82\left(-12.3,\;-1.65\right)\cdot d+14.0\left(12.7,\;15.8\right)\right], \end{array}$$ (5)

where *d* is the euclidean distance between the mid-grey point an the RGB sample, and the values in parentheses represent the lower and upper boundaries of the 95% confidence interval for the estimated coefficient.

**Fig 5:**
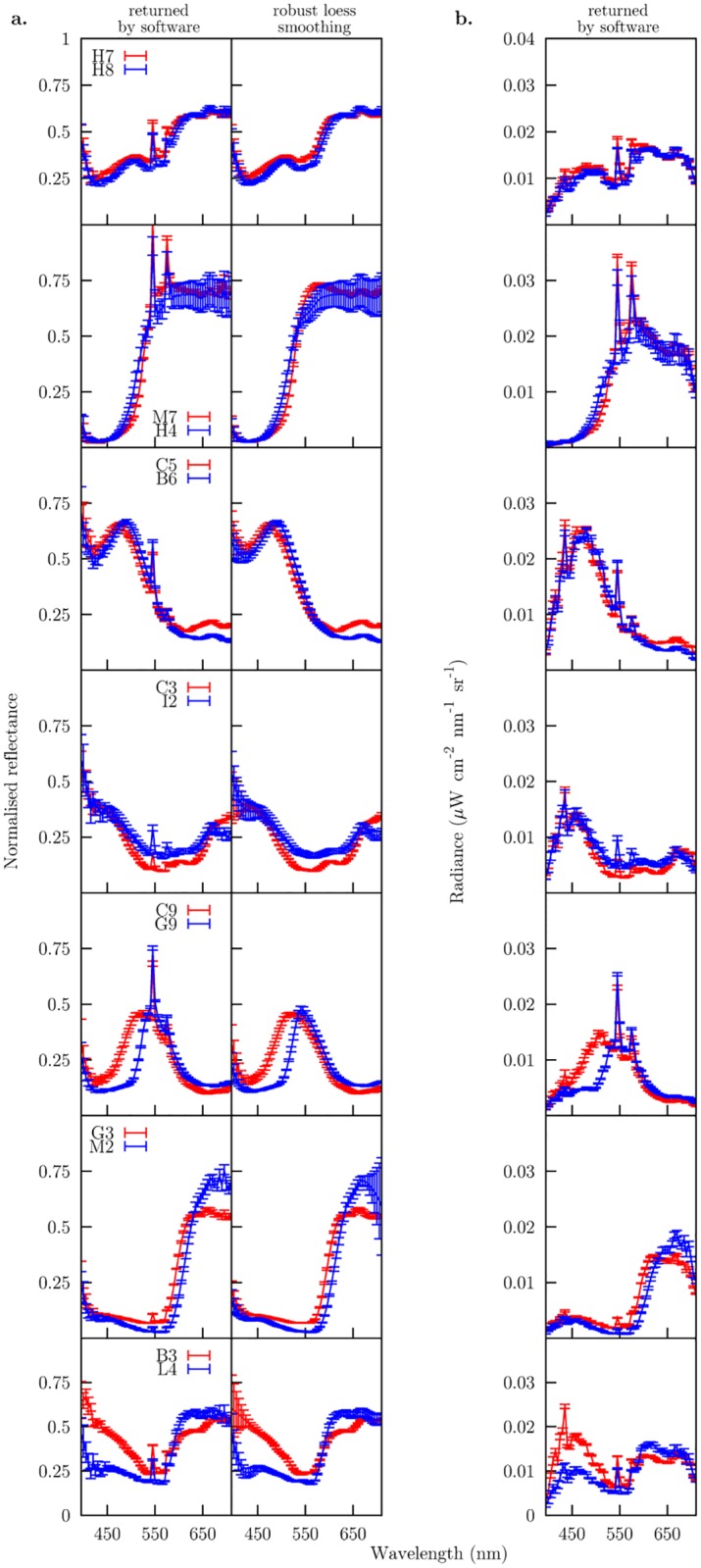
Mean reflectance (panel a.) and radiance (panel b.) spectra recovered from 10 pixel samples of an hyperspectral image cube containing each one of the colour targets in Fig 1. Reflectance spectra in first column of panel a. correspond to the radiance spectra in panel b. after calibrating the radiance responses against a spectrally flat, achromatic surface included in the hyperspectral image cube. Second column in panel a. depicts the result of performing a robust local regression (loess) with a 0.1 span parameter to smooth the peaks observed at about 435 and 545 nm.

Mean reflectance and radiance spectra reconstructed from the 10 pixels sampled from the image hypercube containing the different colour targets are presented in Fig 5. Reconstructed radiance spectra from most of the colour samples present two characteristic peaks at about 545 and 580 nm (Fig 5 panel b) with a secondary peak observable in some samples at 435 nm. Peaks were still present in the reflectance spectra obtained after calibrating the hyperspectral image cube against a spectrally flat surface, suggesting a possible effect of sensor saturation or clipping (Fig 5 panel a). To reduce the potential effect of the peaks in the subsequent colorimetric calculations, we smoothed the reconstructed reflectance spectra by means of a robust local regression (loess) with a 0.1 span. Results from the smoothing operation are depicted in Fig 5 panel a along with the reflectance spectra as returned by the hyperspectral camera software.

### Accuracy of the spectral reconstruction and chromatic discrimination power attainable by the two imaging systems

**Fig 6:**
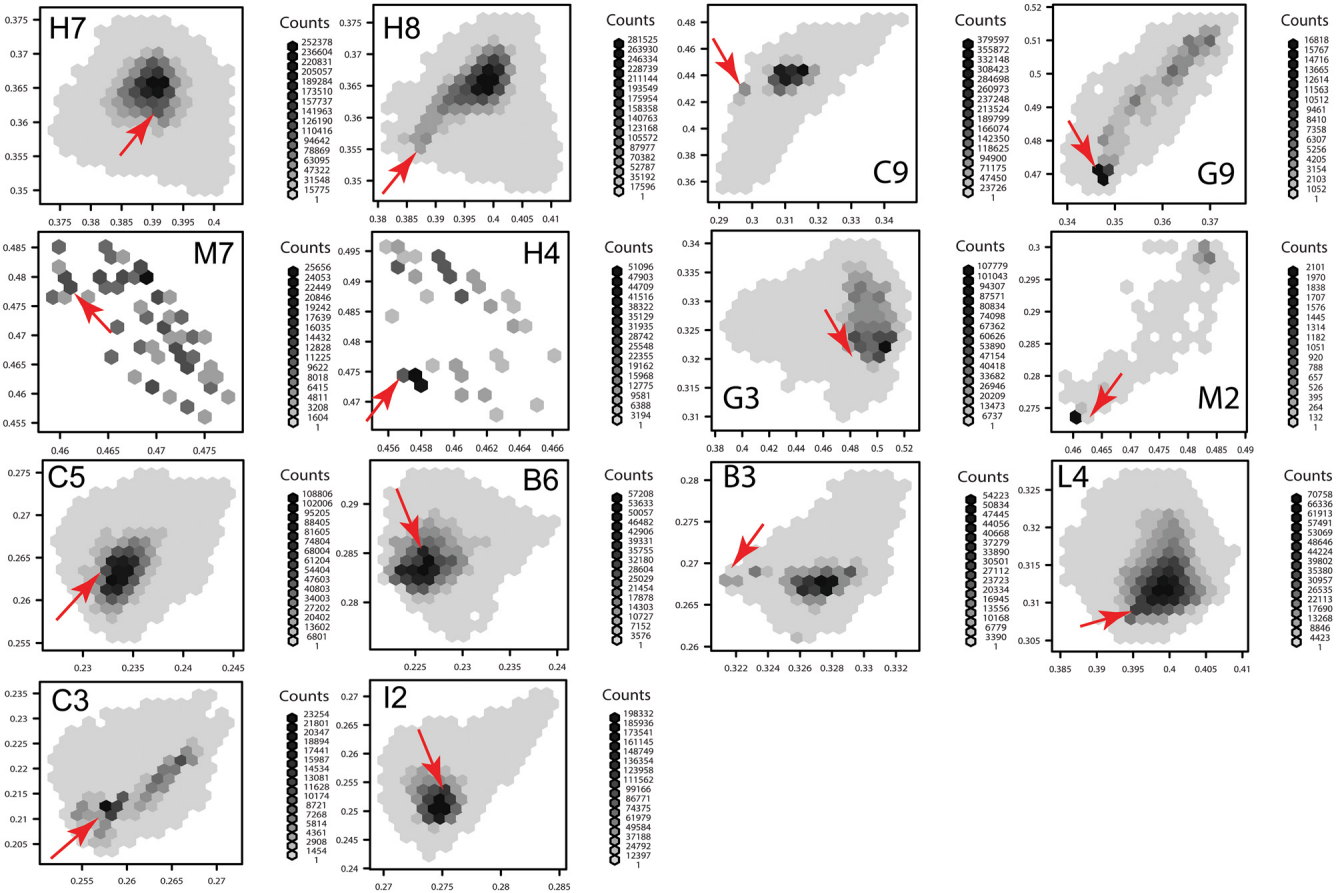
Density scatter plot expressed as hexagonal bins [108] summarising the frequency of chromaticity values obtained from the metamer sets reconstructed from 10 camera response triplets corresponding to each of the 14 colour samples in Fig 1. The number of metamers resulting in the same chromaticity values is represented by grey shades as indicated under the label ‘counts’ for each colour sample. The red arrow on each panel indicates the chromaticity coordinates obtained from the measured reflectance spectrum (panels b and c in Fig 1), and presented in Table 2.

Density scatter plots were constructed from the chromaticity coordinates obtained from the metameter set reconstructed from RGB camera responses (Fig 6). These plots revealed that even though the metamer set reconstructed from any given sample always contained a reflectance spectrum equal to that measured by spectrophotometry, its position within the set changed in an aleatory manner for each colour. Moreover, the distributions of the chromaticity points corresponding to the reconstructed metamer sets presented shapes different from the (bivariate) normal distribution, with bimodal and non-continuous distributions in some cases (Fig 6).

As the goal of spectral reconstruction is recovering the spectrum from a given pixel location within an image rather than measuring it with a spectrophotometer, the most parsimonious solution in the case of the RGB camera was to assume that all metamers have the same probability of being the spectrum corresponding to a measured point sample. Therefore, we expressed the result of the spectral reconstruction process from RGB responses not as a single chromaticity value, but as the entire area encompassed by the chromaticity area (CA).

**Fig 7:**
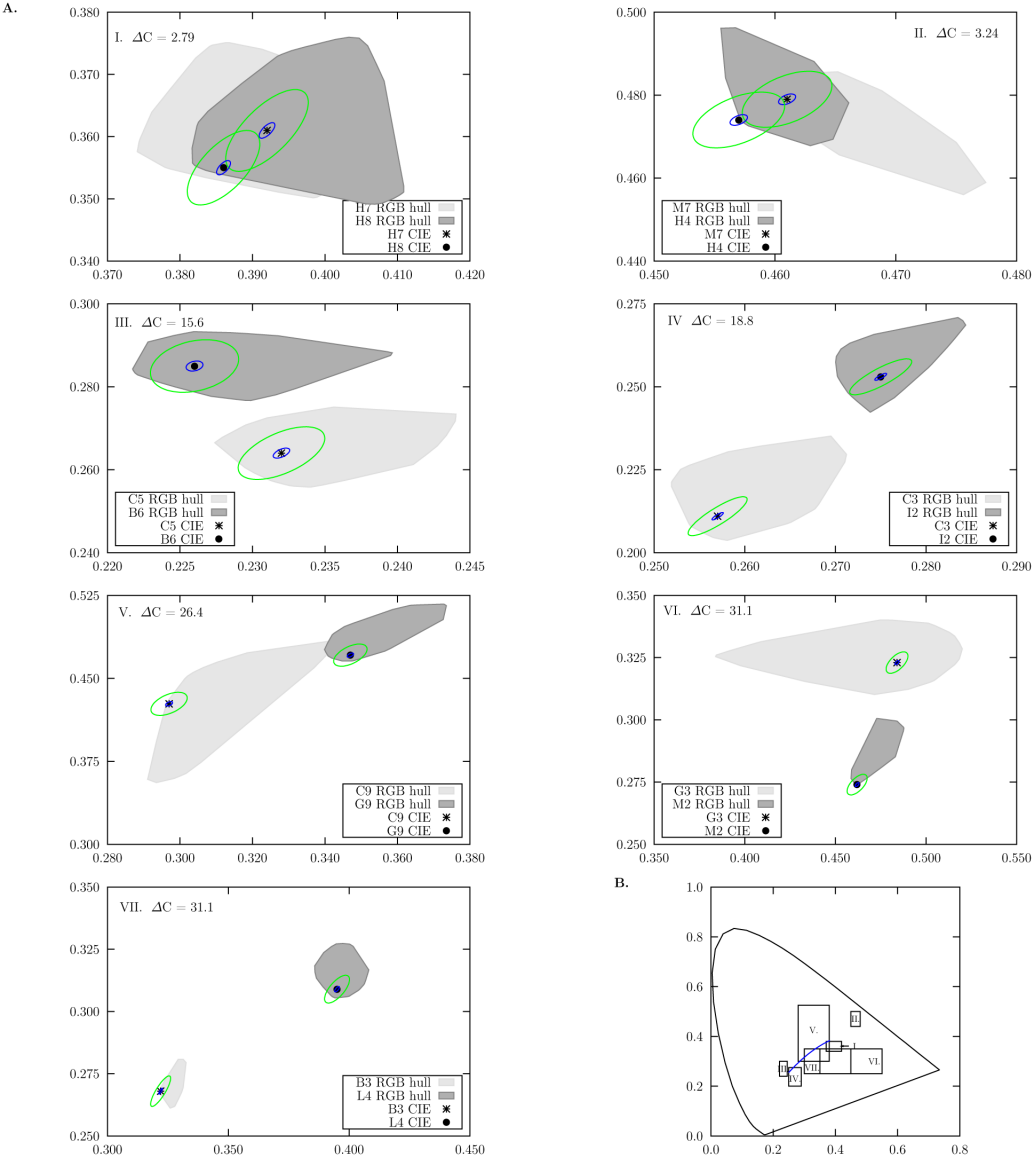
Chromaticity areas (CAs) corresponding to the convex hull of chromaticity coordinates calculated from the metamer sets reconstructed from 10 *ρ*_RGB_ responses of the different colour samples in Fig 1 recorded with an RGB camera. A. Panels correspond to each one of the seven different colour pairs used in our experiment; colour difference values (ΔC) between the pair members are included on each panel. Shaded areas correspond the the CA’s for each colour sample in a sample pair and their intersection represents the confusion region (ARC) expected for a given sample pair. Chromaticity coordinates calculated from measured reflectance spectra are indicated by the (*) and (●) markers. Ellipses represent MacAdam’s [48] (blue) and Newhall [50] (green) colour-difference thresholds calculated for the chromaticity coordinates obtained from measured spectral data. Both ellipses are drawn at their actual scale. B. Regions in the 1931 CIE Chromaticity Diagram covered by the *x* and *y* axis of the panels in A. Variations in area size are due to differences in scale required for plotting the two colours making up each sample pair.

**Fig 8:**
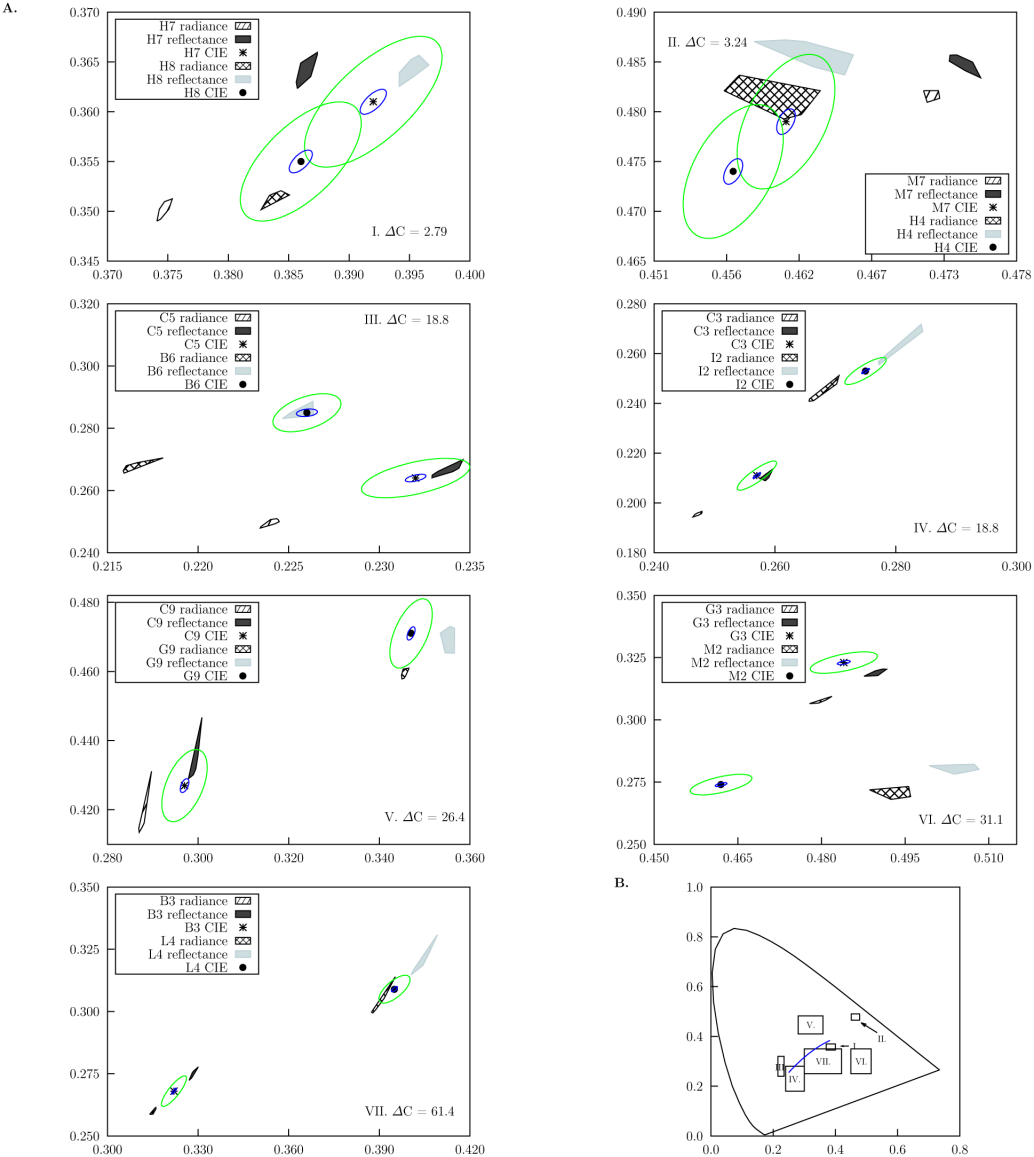
Chromaticity areas (CAs) corresponding to the convex hull of chromaticity coordinates calculated from the radiance and smoothed reflectance spectra reconstructed from 10 pixel responses in the hyperspectral image cube containing the hyperspectral camera responses for the different colour samples in Fig 1, and assuming a mercury discharge lamp as light source (Fig 2). A. Panels correspond to each one of the seven different colour pairs used in our experiment; colour difference values (ΔC) between the pair members are included on each panel. Patterned areas correspond to CA’s calculated from reconstructed radiance spectra, whilst solid shaded areas to CAs calculated from smoothed reflectance spectra for the two colours of each sample pair. Note that confusion regions were not obtained from CAs reconstructed from the hyperspectral camera responses. Chromaticity coordinates calculated from measured reflectance spectra are indicated by the (*) and (●) markers. Ellipses represent MacAdam’s [48] (blue) and Newhall [50] (green) colour-difference thresholds calculated for the chromaticity coordinates obtained from measured spectral data. Both ellipses are drawn at their actual scale. B. Regions in the 1931 CIE Chromaticity Diagram covered by the *x* and *y* axis of the panels in A. Variations in area size are due to differences in scale required for plotting the two colours making up each sample pair.

Sizes of chromaticity areas corresponding to the ten *ρ*_*RGB*_ camera responses are presented in Table 2, along with the CAs calculated from the ten radiance and smoothed reflectance spectra reconstructed from the hyper spectral image cube. CAs were also plotted in the 1931 CIE chromaticity space in Figs 7 and 8.

**Table 2:** Colorimetric properties of samples as chromaticity areas: Chromaticity coordinates corresponding to reflectance spectra measured for each sample constituting the colour pair samples used for the experiment (third column) and chromaticity areas for the spectra reconstructed with an RGB (fourth column) and a hyperspectral camera (fifth column).

Pair	Sample	Chromaticity		RGB camera CA	Hyperspectral camera CA	
		*x*	*y*		radiance	reflectance
I	H7	0.392	0.361	5.06 × 10^−4^	1.15 × 10^−6^	2.90 × 10^−6^
	H8	0.386	0.355	5.44 × 10^−4^	1.53 × 10^−6^	3.51 × 10^−6^
II	M7	0.461	0.479	2.62 × 10^−4^	1.05 × 10^−6^	2.22 × 10^−6^
	H4	0.457	0.474	1.89 × 10^−4^	1.73 × 10^−5^	1.21 × 10^−5^
III	C5	0.232	0.264	2.19 × 10^−4^	1.26 × 10^−6^	2.96 × 10^−6^
	B6	0.226	0.285	1.90 × 10^−4^	2.60 × 10^−6^	4.28 × 10^−6^
IV	C3	0.257	0.211	4.01 × 10^−4^	1.24 × 10^−6^	3.23 × 10^−6^
	I2	0.275	0.253	2.14 × 10^−4^	1.04 × 10^−5^	1.77 × 10^−5^
V	C9	0.297	0.427	2.80 × 10^−3^	8.63 × 10^−6^	1.17 × 10^−5^
	G9	0.347	0.471	9.28 × 10^−4^	2.80 × 10^−6^	1.87 × 10^−6^
VI	G3	0.484	0.323	2.58 × 10^−3^	2.22 × 10^−6^	4.79 × 10^−6^
	M2	0.462	0.274	4.15 × 10^−4^	2.34 × 10^−5^	2.08 × 10^−5^
VII	B3	0.322	0.268	1.24 × 10−4	1.89 × 10^−6^	2.90 × 10−6
	L4	0.395	0.309	3.50 × 10^−4^	1.03 × 10−5	1.81 × 10^−5^
Average chormaticity area Chromaticity area standard error				6.95 × 10^−4^±6.23 × 10^−5^	6.13 × 10^−6^±5.03 × 10^−7^	9.00 × 10^−6^±5.13 × 10^−7^

Chromaticity values were calculated from tristimulus values calculated from Eq (1) using the CIE 1931 colour matching functions.

CAs corresponding to the spectra reconstructed from the hyperspectral camera responses were significantly different from those obtained from the RGB responses (*F* = 102, *P* < 0.001, Table 2). Planned comparison following the omnibus ANOVA test evidenced a significant difference between the area of the RGB CAs and the mean CAs reconstructed from the hyper spectral image cube (*t* = −72.1, *P* < 0.001). However no significant difference was found between the CAs recovered from the radiance and smoothed reflectance spectra (*t* = −1.73, *P* = 0.092).

**Fig 9:**
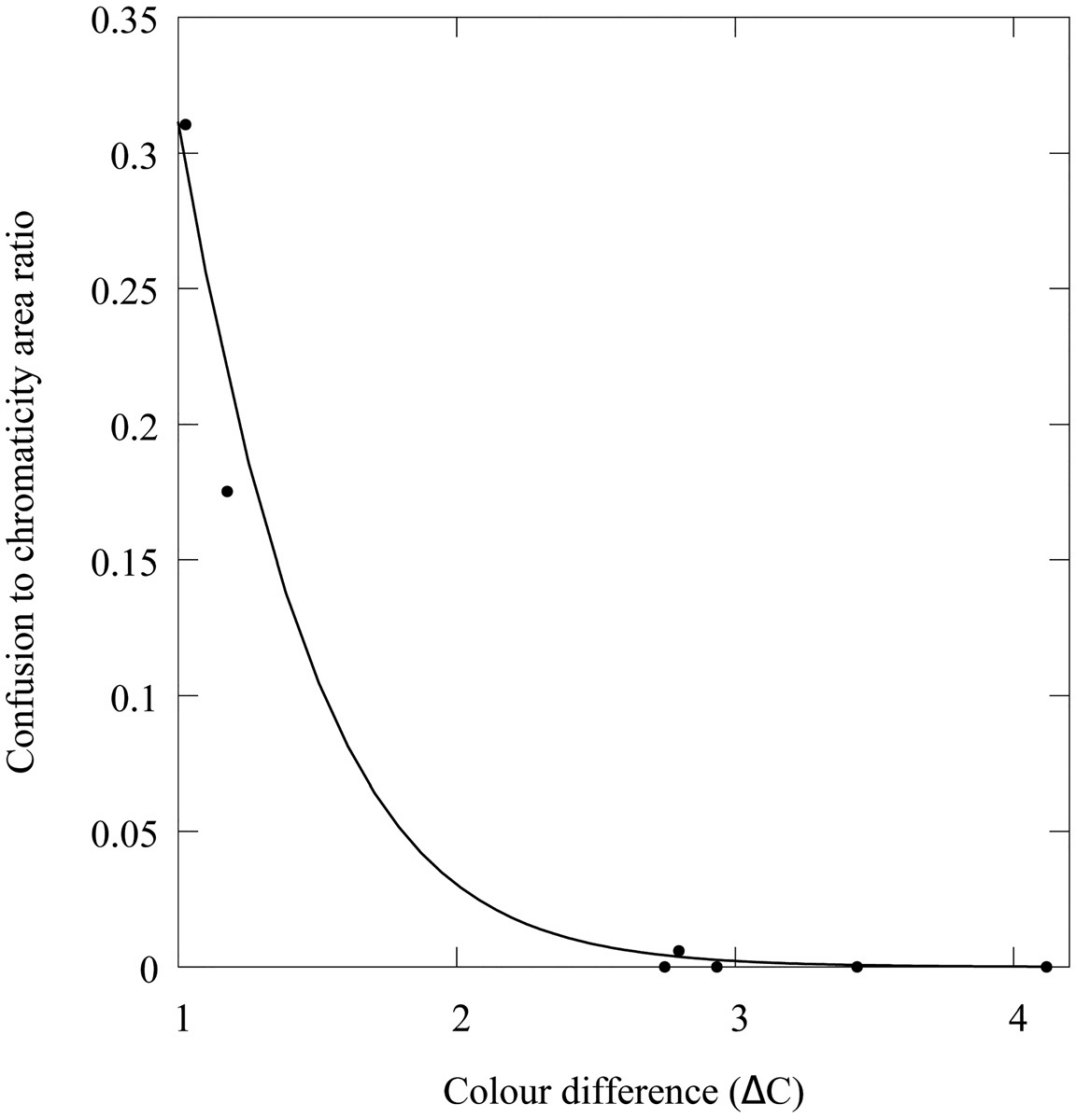
Effect of colour difference (ΔC) in the size of the confusion region for the chromaticity areas corresponding to the metamer sets recovered from the seven colour pairs in Fig 1. The confusion region for each sample pair is defined as the ratio of intersected area to the sum of the individual chromaticity areas of the two colour samples constituting a pair. Chromaticity areas correspond to those displayed in Fig 6. Solid line represents the best fit of an exponential model of the form *y* = *y*_0_ exp−(*bx*) (Eq 6) resulting from a logistic regression using a logit link function.

No confusion areas (ACR) were obtained for any of the CA obtained from either the radiance or smoothed reflectance spectra reconstructed from the hyperspectral camera responses (Fig 8). However we found a significant effect of colour difference (*χ* ^2^ = 1490, *P* < 0.001) on the percentage of ACR shared by the CAs reconstructed from the RGB camera responses. Our data suggest an exponential drop in ACR with increasing colour difference between the samples (Fig 9) described by:

$$\begin{array}{c} ACR=\exp[-2.66(-2.92,\;-2.42)\cdot \Delta \text{C}+1.85(1.55,\;2.18)], \end{array}$$ (6)

where ΔC is the chromatic difference between the two colour samples constituting the pair. Values in parentheses represent the lower and upper boundaries of the 95% confidence interval for the estimated coefficients. Fig 9 depicts the fitted model along with the ACR values for the seven colour pair samples used in our experiment. Data show less than 1% ACR for samples with ΔC > 15. The model predicts about a ΔC > 3.3, about 2 JND, to reduce the ACR percentage to less than 1%.

## Discussion

Spectrophotometry is the most objective and accurate tool available for recording the spectral signature of any given colour sample [24] and allows for data reanalysis using improved or corrected colour discrimination or perception models once more information is made available. Despite the benefit of this objectivity, the recording of spectrophotometric data is limited to point samples which may underestimate the spectral and spatial variability produced by the various properties of the particular elements (patches) comprising natural patterns including colour, shape and texture [19]. This has practical consequences as many of the animals used as model systems for the study of colour evolution possess various combinations of colours in close proximity, and can use multiple mechanisms to produce colour [109–111]. In systems such as these, it is highly unlikely that receivers judge colours in isolation of one another. For this reason a method allowing an effective and practical measurement of reflectance spectra at multiple points within a sampling grid is highly desirable; digital imaging provides such a solution.

Our results show that the degree of accuracy with which the signal spectra can be reconstructed from camera responses is dependent on the number of sensors available in the system (Table 2). In particular, the RGB camera equipped with just three colour channels was unable to accurately discern between samples with a chromatic difference of about 2 JND (Fig 9). This result suggests that RGB cameras are better suited for large-scale examinations in colour patterns such as between-species comparisons (e.g., [112]). Nevertheless, the discrimination ellipses calculated from Newhall’s results for discrimination of small colour differences by 3 observers [50], also intersect for these colour samples (Fig 7). This suggests that differentiating between very similar colours is indeed a difficult task for trichromatic systems.

Results obtained from the RGB camera are in accordance with our stated hypothesis, which predicts a low colour discrimination power with systems with just few sensors as expected from the Principle of Univariance [17]. However, whether animals possessing three or four different photoreceptor classes can unambiguously discern between such small colour differences is a topic worth further investigation. For example, colour discrimination experiments with bees and hawkmoths [65, 113, 114] have evidenced smaller discrimination thresholds than those predicted by models based on spectral sensitivity functions and spectrophotometric data [77].

In contrast to the RGB system, the hyperspectral camera system always resolved spectral differences between the two colour samples irrespective of their similarity (Fig 7). This level of resolution is provided by the 128 sensors available to recover the spectral signals measured at 64 discrete intervals [38]. Rather than observing compact clusters of ten overlapping chromaticity coordinates for each colour target, we obtained chromaticity areas of varying shape and size (Fig 7, Table 2).

Interestingly, some of the obtained chromaticity areas (CAs) defined by the hyperspectral approach are larger than the discrimination ellipses proposed by MacAdam [48] and Newhall [50] (Fig 7). This suggests the existence of potentially perceivable colour differences within some of the colour sample targets at least for a human observer. Such a chromatic variability has also been reported for flowers based on quantitative analysis on linearised RGB images [19], supporting the idea that observed chromatic variability is not only the result of different sources of image noise (e.g. shot, thermal, quantisation present in the different colour channels [115, 116]), but an intrinsic property of natural and man-made colour surfaces.

Spatio-chromatic variability has been greatly overlooked until recently due in part to the inherent difficulty of its accurate measurement from point samples [117]. However exploring spatiochromatic variability in biological samples is important as variation can be a result of texture, volume and micro structures in plants and animals [109, 110, 118, 119], especially because such variation is not uniformly distributed within the biological sample. In addition, these microstructures may change through wear and ageing [120] increasing the variation in the signal produced. Understanding how these factors change would provide greater insight into the evolution of colour as a signal. For example, recent imaging revealed significant amounts of within-subject chromatic variations, providing interesting insights into the way observers other than humans may perceive signals produced by plants [19] and animals [40] for communication and camouflage. RGB systems may thus not be ideal for examination of colour signalling in multi-coloured species that contain multiple patches, in particular when the objective of the study is to identify and describe the different chromatic elements comprising them.

Characterisation and quantification of the spatio-chromatic variability present in a sample is an example of a biological problem well suited for hyperspectral imaging, as it allows for a precise quantitative estimation of within-subject and intra-specific colour variation. Such explorations can provide greater insight into plant-insect visual communication [121, 122], the role of relative variance in multi-coloured patches in fitness [123], and the role of ageing on colour expression [124]. Likewise, the study of animal camouflage in the context of classic background matching, where both target and background share similar values in colour and brightness [24], can benefit from hyperspectral imaging which allows precise spectral measurements on small targets and natural backgrounds displaying complex patterns with various colours, shapes and designs. However, an important point to consider when using these devices is the extent to which a hyperspectral camera serves as a model for animal colour discrimination. Currently, just a few organisms which could be regarded as possessing a ‘hyperspectral-type visual system’, have been reported to actively use more than four photoreceptors for colour discrimination tasks [125–128]. However, many animal species tested to date show a range of species-specific photoreceptor distribution [129–132], and so mapping complex stimuli with hyperspectral cameras can cater for new discoveries and guidance on how to model other important effects such as colour constancy.

**Fig 10:**
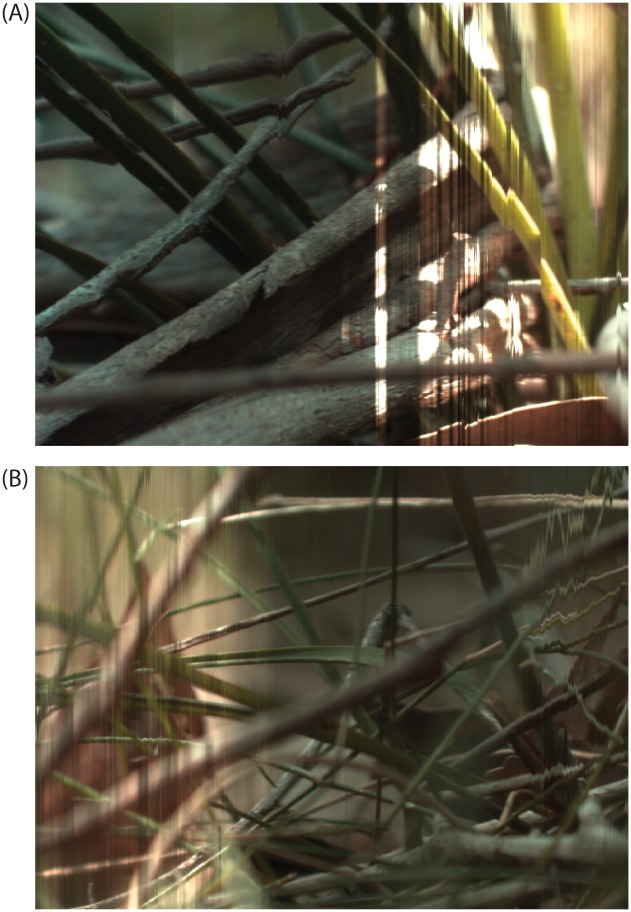
Hyperspectral image scans generated outdoors under a) partially cloudy and b) windy conditions using sunlight as the only illumination. a) Passing clouds blocked the sun during image recording resulting in various underexposed image regions. Similarly, darkened portions of (b) are a result of overhead leaf cover shading direct sunlight at the end of the scan as the wind increased their movement. Integration time for both scans was set to 50.00 ms.

Hyperspectral cameras offer new possibilities for the study of colour constancy in natural contexts. Hyperspectral cameras possess narrow-band sensors in a number higher than the one required for optimally sampling natural spectra, characteristics closely resembling the ideal system for attaining a perfect colour constancy [133]. To date, most of the advances in this topic have been done based on theoretical models [53, 133–137], and the hyperspectral imaging principle opens a new perspective in the study of colour constancy by allowing the recording and analysis of natural scenes under various types of ambient illumination. However, exposure times required by hyperspectral imaging may limit the extent to which ambient light variations can be accurately allowed for with these devices. Indeed, the dynamic nature of other environmental factors (e.g. cloud cover) can make it difficult to capture a whole scene under exactly the same lighting conditions in the field (Fig 10) since natural lighting conditions often change rapidly in short spatial distances [53]. Despite this disadvantage, hyperspectral cameras may allow for new opportunities to explore evolutionary questions regarding colour investment. For example, does variation in illumination and/or background alter the relative benefit of investing in colour traits in different animals? Such possibilities have been explored for birds displaying in forests and competing for patches of specific illumination to potentially maximise the communication of colour signals [138], but currently the answer remains unknown for many animals operating in complex natural environments.
